# High prevalence of workplace violence among nurses working at public health facilities in Southern Ethiopia

**DOI:** 10.1186/s12912-015-0062-1

**Published:** 2015-03-03

**Authors:** Mathewos Fute, Zelalem Birhanu Mengesha, Negash Wakgari, Gizachew Assefa Tessema

**Affiliations:** Sidama Zone Social and Labour Affairs, Southern Nation, Nationality and People Regional State, Hawassa, Ethiopia; Department of Reproductive Health, Institute of Public Health, University of Gondar, Gondar, Ethiopia; School of Nursing and Midwifery, College of Medicine and Health Sciences, Hawassa University, Hawassa, Ethiopia

**Keywords:** Public Health Facilities, Nurse, Workplace Violence, Ethiopia

## Abstract

**Background:**

The rising rate of workplace violence in health care facilities has become a major problem for health care providers including nurses. However, evidences are lacking in Ethiopia particularly in the study area. The aim of this study is to assess the prevalence and associated factors of workplace violence among nurses working at health care facilities in Hawassa City Administration, Southern Ethiopia.

**Methods:**

An institution-based cross-sectional study was conducted on 660 randomly selected nurses working at public health facilities in Hawassa City Administration in April 2014. A pre-tested and structured questionnaire was used to collect the data. Data were entered using EPI-Info and exported to SPSS for further analysis. Descriptive statistics were done. Logistic regression analyses were used to see the association between different variables and the outcome variable. Odds ratios with 95% Confidence Interval (CI) were computed to determine the presence and strength of the association.

**Results:**

In this study, the prevalence of workplace violence was 29.9% [95% CI: 26.5, 33.5)] of which physical violence accounted for 36 (18.22%), verbal abuse for 172 (89.58%) and sexual harassment for 25 (13.02%). Female sex [AOR=2.00, 95% CI: (1.28, 2.39)], short work experience [AOR=8.86, 95% CI: (3.47, 22.64)], age group of 22–25 [AOR=4.17, 95% CI: (2.46, 7.08)], age group of (26–35) [AOR=1.9, 95% CI (1.16, 3.1)], work in emergency [(AOR=4.28, 95% CI: (1.39, 4.34)] and work in the Inpatient Department [(AOR=2.11, 95% CI: (1.98, 2.64)] were the factors positively associated with workplace violence.

**Conclusions:**

A significant proportion of nurses faced violence while providing care at in public health facilities. Being female, younger age, short work experience, and assignment in emergency and inpatient departments were positively associated with workplace violence. Policy makers and stakeholders should focus on workplace violence prevention strategies.

## Background

Workplace violence against health care workers is a common and widespread phenomenon. According to the World Health Organization (WHO), workplace violence where staffs are abused, threatened, or assaulted in circumstances related to their work and while commuting to and from work, involved explicit or implicit challenges to their safety, well-being, or health. The violence affects all work categories and takes place at various settings [1-3]. About 25% of violent accidents at work occur in the health sector, and more than 50% of health workers have already experienced violence [4,5]. Each year, more than 1.6 million people worldwide lose their lives in relation to violence, and many more are injured and suffer from physical and non-physical health problems [6]. Violence related injury is the second leading cause of occupational injury and accounts for 16% of the more than 6.5 million acts of violence experienced by individuals [4].

According to the International Labour Office (ILO) report, nurses faced more violence than other health care workers [7,8]. Nurses as front-line care providers serve in a wide variety of settings caring for individuals who face all types of trauma, suffering, and life-altering events [9-11].

Exposure to violence while carrying out duties adversely affect nurses and may lead to loss of concentration, inattention to ethical guidelines, commuting mistakes, absence from shifts, repeated absenteeism, inattention to patients, reduction in job satisfaction, dislike of job, and refusal to work in stressful wards. As a consequence of experiencing violence in the workplace, a nurse may decide to transfer to another section with in the same health care facility, or may give up nursing altogether. This may result in significant additional costs on treatment centers and the community [6,12,13]. Moreover, the consequences of workplace violence in the health sector have a significant impact on the effectiveness of health systems, especially in developing countries.

However, there is a lack of evidence to support this concern due to the absence of information on the prevalence of workplace violence among nurse professionals in developing countries, like Ethiopia. Therefore, this study is meant to assess the prevalence and associated factors of workplace violence among nurses working at public health facilities in Hawassa City administration, Southern Ethiopia.

## Methods

### Study design, sample, sampling technique and setting

An institution based cross-sectional study was conducted in April 2014 among nurses working in public health facilities in Hawassa City Administration, Southern Ethiopia. Hawassa city is located in Southern Nations, Nationalities, and Peoples’ Regional State, Southern Ethiopia, 275 km from Addis Ababa. All public health facilities in Hawassa City Administration (2 governmental hospitals and 10 health centers) were included in the study. The sample size was calculated using the single population proportion formula and by considering the assumptions, the proportion (p) of 50%, 95% confidence level of Za/2=1.96, 4% margin of error, and a non-response rate of 10%. Hence, the total calculated sample size was 660. There were a total of 1,687 health workers in the public health facilities of study area of whom 850 (more than half) were nurses. All nurses working in the public health facilities of the city were considered as the study population. The study participants were selected by proportional allocation, based on the number of nurses that the respective health facilities contained and K numbers of the participants were drawn by the lottery method.

$$ \mathrm{K}=\frac{\mathrm{required}\ \mathrm{sample}\ \mathrm{size}(660)\times \mathrm{number}\ \mathrm{of}\ \mathrm{nurses}\ \mathrm{in}\ \mathrm{respective}\ \mathrm{health}\ \mathrm{facility}}{\mathrm{Total}\ \mathrm{number}\ \mathrm{of}\ \mathrm{nurses}\ \mathrm{in}\ \mathrm{the}\ \mathrm{study}\ \mathrm{area}\ (850)} $$

### Data collection instrument

The data were collected using a structured, pretested, and self-administered questionnaire that was adapted from the International Labour Office (ILO), International Council of Nurses (ICN), World Health Organization (WHO), and Public Services International (PSI) [2]. It was reviewed to suit the local condition. The questionnaire was initially prepared in English, then translated to the working language (Amharic) and back to English in order to ensure conceptual consistency. The questionnaire consisted of 4 parts: Part I enquired about the socio-demographic characteristics of the nurses; part II assessed the most recent physical abuse and nurses’ reaction for such episodes in the past 6 months prior to the study period. Part III focused on verbal abuse and the nurses’ reactions to these events Part IV, the last section, enquired about the sexual harassment and nurses’ reaction at that scene. Workplace Violence (WPV) was regarded when the study participants experienced at least one type of violence such as physical violence, verbal abuse, or sexual harassment in circumstances related to their work in the past six month.

Verbal abuse was defined as shouting at, degrading and showing lack of respect for someone’s worth and dignity; hence, nurses were asked to check whether, they had experienced any of the verbally abusive behaviors listed from 4 different sources (co-workers, patients, and patients’ relatives). Physical violence was defined as hitting, biting, throwing objects, strangling, pushing around, kicking, dragging on the floor, pushing against the wall, beating with a stick, threatening an individual with a gun, a knife, or any kind of weapon. Sexual harassment involved attempts to establish or force sexual relations, to threaten someone into having sex (sexual blackmail), and to offering money, gifts, or privileges in exchange for sexual favors. Pretesting of the study tool was conducted on 20 nurses working at Yirgalem Hospital. Finally, the necessary modifications and adjustments were made on the tool prior to the actual study. Twelve data facilitators watched over by three supervisors were involved in the data collection.

### Data analysis

The collected copies of the questionnaire were checked manually for its completeness, coded and entered into the EPI-Info version 7 statistical packages, and exported to SPSS version 20.0 for further analysis. Descriptive statistics were done. Both bivariate and multivariate logistic regression analyses were used to determine the association of each independent variable with the dependent variable. Odds ratio with their 95% confidence intervals were computed to identify the presence and strength of association, and statistical significance was declared if p < 0.05.

### Ethical considerations

Ethical clearance was obtained from the Research and Ethical Review Committee of the Institute of Public Health, College of Medicine and Health Sciences at the University of Gondar. Official letters were also submitted to respective public health facilities. The purpose and importance of the study were explained and written consent was secured from each participant. Confidentiality was maintained at all levels of the study. Participant involvement in the study was voluntary and those who were unwilling and wanted to quit their participation at any stage were informed to do so without any restriction.

## Results

### Socio-demographic characteristics of respondents

A total of 642 respondents were included in the final analysis making a response rate of 97.3 percent. Eleven of the copies rejected were due to data incompleteness of relevant variables, while seven participants did not return the questionnaire to the data facilitators. Nearly two thirds (62.93%) were females, and half (50%) were married. The mean age of the participants was 30.23 years (SD=6) and about 43% were between the age group of 36 and 52 years. Out of the total study subjects, about 42% were protestant. With regard to their education, about 56% had bachelor’s degree in nursing (Table 1).

**Table 1:** **Socio-demographic characteristics of nurses working at public health facilities in Hawassa city, Southern Ethiopia, April, 2014 (n=642)**

**Variables**	**Frequency**	**Percentage**
**Sex**		
Male	238	37.1
Female	404	62.9
**Age category**		
≤25	146	22.7
26-35	224	34.9
36–52	272	42.4
**Marital status**		
Single	288	44.9
Married	321	50
Separated /Divorced/widowed	33	5.1
**Religion**		
Protestant	300	46.7
Orthodox	258	40.2
Muslim	63	9.8
Others	21	3.3
**Ethnicity**		
Sidama	197	30.7
Hadiya	144	22.4
Guraghe	146	22.7
Wolayita	67	10.4
Amhara	88	13.7
**Educational status**		
Diploma	286	44.5
Bachelor (BSc)	356	55.5

### Workplace characteristics of nurses

About 62% of the participants were working at hospitals, and nearly forty four percent of these were working at the Outpatient Department (OPD), about sixty one of the participants reported that they were working on shifts.. About one-third (32.39%) of the study subjects had less than 5 years of experience. Nearly three quarters of them reported that they were worried about violence in their workplaces; and more than half (51.2%) reported that there were no procedures for reporting violence incidents in their workplaces (Table 2).

**Table 2:** **Organizational and work characteristics of nurses working at public health facilities in Hawassa city administration, Southern Ethiopia, April, 2014 (n=642)**

**Variables**	**Frequency**	**Percentage**
**Job-position**		
Ward-head/nurses head	55	8.6
Staff/service provider	587	91.4
**Facility type**		
Public Hospital	395	61.5
Health Center	247	38.5
**Reporting procedure**		
Available	313	48.2
Not available	329	51.2
**Clinical setting**		
Outpatient department	281	43.8
Inpatient department	233	36.3
Emergency department	128	19.9
**Experiences**		
1–5	208	32.39
6–10	186	28.97
11-15	173	26.94
16–26	75	11.68

### Prevalence of workplace violence

**Figure 1:**
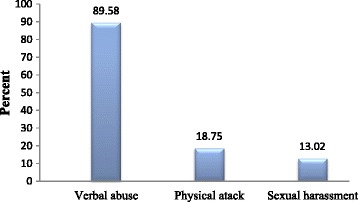
**Type of workplace violence against nurses working at health care facilities during past 6 months.**

About one-third (29.9%) [95% CI: (26.5, 33.5)] of the nurses experienced at least one incident of the various forms of workplace violence (physical, verbal, and sexual harassment) in the past 6 months. Verbal abuse was the most common (89.6%) form of violence encountered by nurses followed by physical violence (18.8%), and sexual harassment (13%). Moreover, about eighteen percent were victims of at least two form of workplace violence. Nearly one-third (32.7%) witnessed violence in the past 6 months (Figure 1). About six percent of the nurses reported that they had been physically attacked, about forty five percent of the incidents were caused by patients. On the other hand, half of the physical violence happened on the evening shift.

### Factors associated with workplace violence

The bivariate analysis showed that there was a statically significant association between workplace violence and sex, age, work experience, clinical setting (department), and shifts. However, in the multivariate logistic regression analysis, sex, age, experience and clinical setting (department) remained to be significant. Those who had 1–5 years of experience had about 9 times higher odds of experiencing workplace violence than those who had 5 or more years of work experience (AOR=8.9, 95% (3.47, 22.63)). Female nurses had two times higher odds of experiencing workplace violence compared to male nurses [AOR=2.0, 95% CI: (1.28, 3.12)].

Occupational characteristics of nurses revealed that the odds of violence against nurses were about four times higher among emergency department workers than those who served in the outpatient departments [AOR=4.3, 95% CI: 4.27 (2.44, 7.50)]. Likewise, the odds of workplace violence were 2 times higher among nurses working at inpatient departments than outpatient departments [AOR=2.1, 95% CI: (1.98, 3.42)]. Nurses with age group of 22–25 had about four times higher odds of experiencing violence compared with those aged 36–52 years [AOR=4.2, 95% CI: (2.46, 7.08)]. Similarly, nurses aged between 26–35 years had about two times higher odds of experiencing violence compared to the 36–52 age group [AOR=1.90, 95% CI: (1.16, 3. 11)] (Table 3).

**Table 3:** **Crude and adjusted odds ratios (OR) and 95% (CI) of factors associated with workplace violence at public health facilities in Hawassa City Administration, April, 2014 (n=642)**

**Variables**	**Workplace Violence**		**OR (95% CI)**	
	**Yes**	**No**	**COR (95% CI)**	**AOR (95% CI)**
**Sex**				
Male	47	191	1	1
Female	145	259	2.3 (1.56, 3.32)	2.0 (1.28, 3.12)
**Age category**				
≤25	81	65	7.0 (4.41, 11.18)	4.2 (2.46, 7.08)
26–35	70	154	2.6 (1.66, 3.96)	1.9 (1.16, 3.11)
36 +	41	231	1	1
**Years of experience**				
1-5	102	106	11.1 (4.60, 26.6)	8.9 (3.47,22.63)
6–10	42	144	3.6 (1.36, 8.27)	3.4 (1.37,9.31)
11–15	42	131	3.2 (1.49, 9.10)	3.3 (1.67, 11.44)
16+	6	69	1	1
**Working department**				
Outpatient	38	189	1	1
Inpatient	85	202	2.1 (1.36, 3.22)	2.1 (1.98, 3.42)
Emergency	69	59	5.8 (3.55, 9.51)	4.3 (2.44,7.50)

## Discussion

This study was the first of its kind to report WPV against nurses working at health care facilities in Ethiopia. Currently, it is among the priority concerns of both industrialized and developing countries [14].

The prevalence of workplace violence on nurses working at public health facilities was 29.9% [95% CI: 26.5, 33.5)]. This finding is similar to findings in USA (30%) [7] and Egypt (27.7%) [15]. However, it is lower than that of a study conducted in Palestine (80%) [16]. This could be due to lack of violence preventing strategies, such as policy/procedures, training, adequate safety measures in Palestinian public health facilities.

In the present study there was a statistically significant relation between age and workplace violence, as the age of health workers increased, the violence committed against them decreased. Young nurses had higher odds of experiencing workplace violence compared to their older counterparts. This finding is in line with those of studies conducted in Saudi Arabia and Taiwan [17,18]. This might be due to young nurses’ lack of ability or experience in dealing with violence, and inadequate safety measures. In addition, this might partly be explained by the fact that older people including health care providers receive due respect in the Ethiopian culture and perhaps elsewhere.

The sex of the study participants had a significant association with workplace violence; females had higher odds of being exposed to workplace violence than males. This is in line with studies conducted in Egypt and Palestine [15,19]. This might be due to traditional thinking that dictates that men are at the top of the hierarchical structure and superior to females. This traditional thinking may explain the more common occurrence of violence on females.

The other finding of this study showed that nurses who had less than six years of service had more odds of experiencing workplace violence than nurses who served sixteen or more years. This result is consistent with the finding in Southern Taiwan [20]. This might be due to the fact that nurses with shorter service years had less experience in dealing or preventing various types of clashes and could not dissolve the possibility of an abuse incident promptly, so they experienced more counts of verbal and physical abuse.

Clinical setting was also associated with workplace violence. Nurses working at emergency departments were facing higher odds of violence compared to those serving in outpatient departments. This finding is similar to that of a study conducted in USA [21]. This might be happened due to as emergency departments are open twenty four hours in the absence of security guards. The other possible justification could be that attendants of patient at emergency clinics were are tempered to aggress due to the stressful environment.

The possible limitation of this study was lack of willingness of the study participants to disclose private information. Recall bias and lack of research in Ethiopian context made comparison difficult.

## Conclusions

A significant proportion of nurses at Hawassa city health care facilities had experienced different forms of violence. The results of the study suggested that violence was a major occupational hazard and public health concern. Female sex, youth age, and short years of work experience had a positive association with the incidence of workplace violence. Policy makers and other stakeholders should focus on the provision of appropriate strategies on workplace violence prevention. The health facilities should also establish health and safety programs for the prevention and management of workplace violence. It is also advisable to provide priority attention to female, and young nurses.
